# A genetic survey of patients with familial idiopathic intracranial hypertension residing in a Middle Eastern village: genetic association study

**DOI:** 10.1186/s40001-024-01800-z

**Published:** 2024-03-25

**Authors:** Eran Berkowitz, Tzipora C. Falik Zaccai, Dana Irge, Inbar Gur, Beatrice Tiosano, Anat Kesler

**Affiliations:** 1 Department of Ophthalmology, Hillel Yaffe Medical Center, 1 Ha-Shalom Street, 38100 Hadera, Israel; 2 Sackler Faculty of Medicine, Tel Aviv University, Tel Aviv, Israel; 3 The Azrielli Faculty of Medicine, Bar Ilan University, Safed, Israel; 4 The Adelson School of Medicine, Ariel University, Ariel, Israel; 5 The Ruth and Bruce Rappaport Faculty of Medicine, Technion, Haifa, Israel; 6 Genetic Institue, Meir Medical center, Kfar Saba, Israel

**Keywords:** Idiopathic intracranial hypertension, GWAS, PTC, Genetic association, Familial

## Abstract

**Background:**

The aim of this study was to determine whether genetic variants are associated with idiopathic intracranial hypertension (IIH) in a unique village where many of the IIH patients have familial ties, a homogenous population and a high prevalence of consanguinity. Several autosomal recessive disorders are common in this village and its population is considered at a high risk for genetic disorders.

**Methods:**

The samples were genotyped by the Ilumina OmniExpress-24 Kit, and analyzed by the Eagle V2.4 and DASH software package to cluster haplotypes shared between our cohort. Subsequently, we searched for specific haplotypes that were significantly associated with the patient groups.

**Results:**

Fourteen patients and 30 controls were included. Samples from 22 female participants (11 patients and 11 controls) were evaluated for haplotype clustering and genome-wide association studies (GWAS). A total of 710,000 single nucleotide polymorphisms (SNPs) were evaluated. Candidate areas positively associated with IIH included genes located on chromosomes 16, 8 (including the *CA5A* and *BANP* genes, *p* < 0.01), and negatively associated with genes located on chromosomes 1 and 6 (including *PBX1*, *LMX1A*, *ESR1* genes, *p* < 0.01).

**Conclusions:**

We discovered new loci possibly associated with IIH by employing a GWAS technique to estimate the associations with haplotypes instead of specific SNPs. This method can in all probability be used in cases where there is a limited amount of samples but strong familial connections. Several loci were identified that might be strong candidates for follow-up studies in other well-phenotypes cohorts.

## Background

Idiopathic intracranial hypertension (IIH), also known as pseudotumor cerebri (PTC), is a disorder of unknown etiology, predominantly affecting obese women of childbearing age [1, 2]. At present, IIH is considered a sporadic disease, although some researchers have suggested that IIH may have a familial component [3]. Few familial occurrences have been recorded [3–10], although, in the Idiopathic Intracranial Hypertension Treatment Trial (IIHTT), 5% of patients stated that other family members were affected with IIH [4]. In other studies, the rate of familial occurrences has been reported between 2.9% and 11% [3–5], which is higher than its rate in the general population [11]. Recently, the NORDIC IIHTT Study Group [10] ran a Genome-Wide Association Study (GWAS) on the IIHTT cohort, using Chips interrogating 538,448 markers to determine whether genetic variants are associated with this condition. The study identified three candidate regions marked by multiple associated single-nucleotide polymorphisms (SNPs) on chromosomes 5, 13, and 14.

Our study cohort comprised Israeli patients and their families affected with IIH residing in a village in northern Israel known for its homogenous population and high prevalence of consanguinity. Several autosomal recessive disorders are common in this village and its population is considered at a high risk for genetic disorders. Severe diseases such as intellectual disability, non-syndromic hearing loss, spinal muscular dystrophy related-disease, primary microcephaly (MCPH), and Basel-Vanagaite–Smirin-Yosef Syndrome have been observed in this population [12, 13]. This population mainly descends from Egyptian ancestors who arrived in the 1800s, together with Bedouin tribes from the Jordan Valley [14]. Many of the village’s original families and descendants have remained in the village, and today encompasses ~15,000 inhabitants. Our aim was to investigate the genetic variants that may be associated with IIH in a population residing in this village, as a high prevalence of IIH patients from this village relative to its size has been observed.

Given the potential small sample size (stemming from this cohort’s special characteristics), using traditional GWAS analysis techniques to analyze the data from this village, may be inappropriate. Discovering a GWAS method to evaluate small cohorts with some genetic similarity, while evaluating and analyzing DNA from patients suffering from IIH in this village, using advanced analytical methods, may offer a possible insight into this disorder and further advance our knowledge of this disease’s possible etiology.

## Methods

This study protocol was approved by the local Institutional Review Board and Ethics Committee of the Hillel Yaffe Medical Center, Israel and the Supreme IRB Committee of the Israeli Ministry of Health (reference number is 030-2018, approved on the 29th of May 2018). Written informed consent was obtained from all study participants. Study subjects were inhabitants of this village recruited from IIH patients referred to the Neuro-Ophthalmology Clinic, Hillel Yaffe Medical Center, Israel, between 2018 and 2020. Controls included healthy first-degree family members also living in this village. All participants underwent a detailed clinical evaluation to diagnose IIH according to the modified Dandy criteria [15]. Controls were examined to exclude those with symptoms or signs of IIH. Buccal swab DNA samples were obtained from the patients and the controls by MyHeritage’s DNA kits. The samples were then shipped to “Gene by Gene” LTD (Houston, TX, USA) for genomic DNA isolation and genotyping.

### Single-nucleotide polymorphism array genotyping

Genome-wide SNP genotypes were obtained for all samples using MyHeritage’s costume array, based on the Ilumina OmniExpress-24 Kit, containing ~710,000 markers (SNPs).

### Ethnicity estimation

Ethnicity of the patients and controls was evaluated by the MyHeritage™ Ethnicity Estimation algorithm, which calculated the percentages for each region from which the subjects’ DNA was inherited.

### Familial relations and consanguinity analysis

To measure familial relations in our cohort of patients and controls, the identity by descent (IBD) MyHeritage’s relative matching algorithm [16] determined the genetic sharing (in centiMorgan) between the participants.

### Associations between SNPs and IIH

**Fig. 1:**
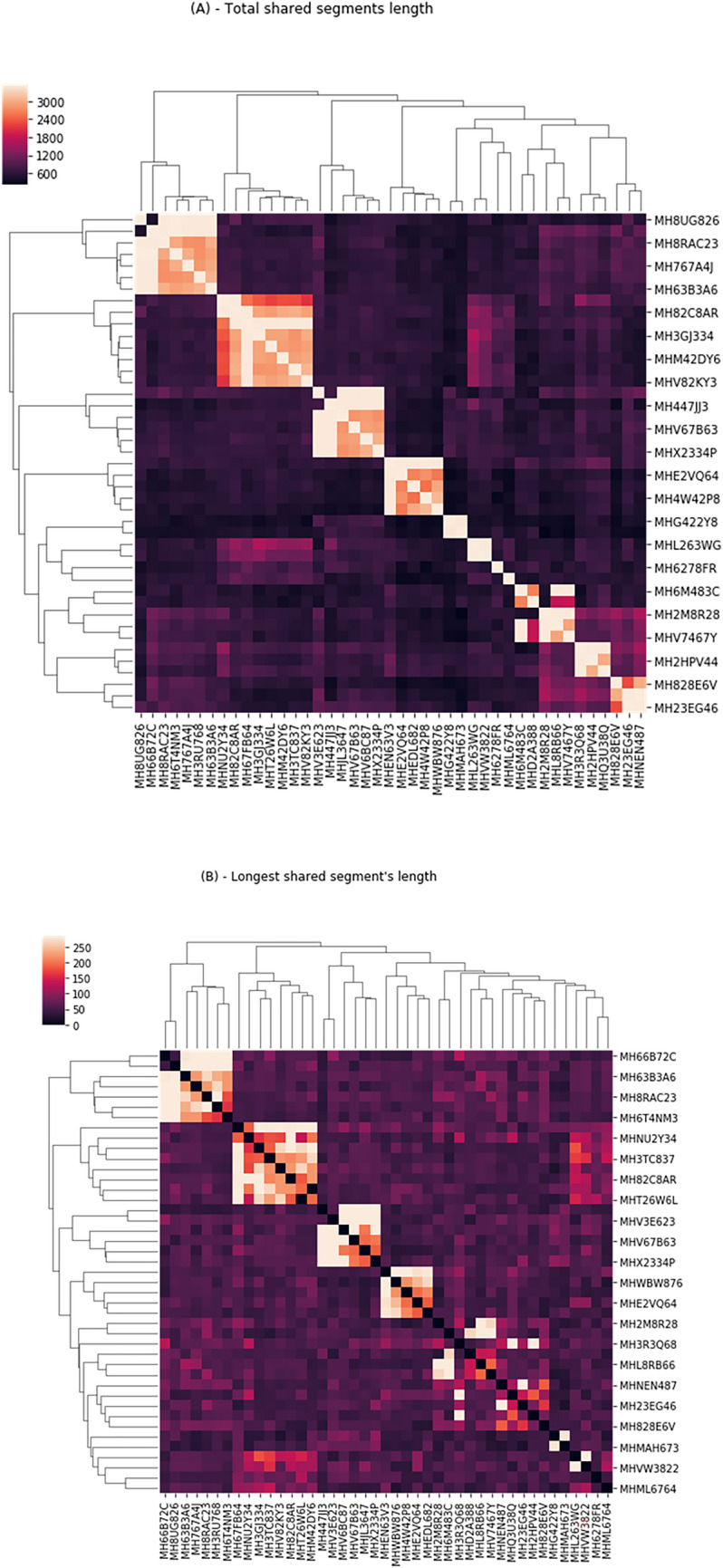
**A** Dendrograms of study participants showing the shared DNA between the individuals of the study. Total shared segment length (cM) between all individuals of the cohort. **B** same as **A** using the length of the longest shared segment (cM)

**Fig. 2:**
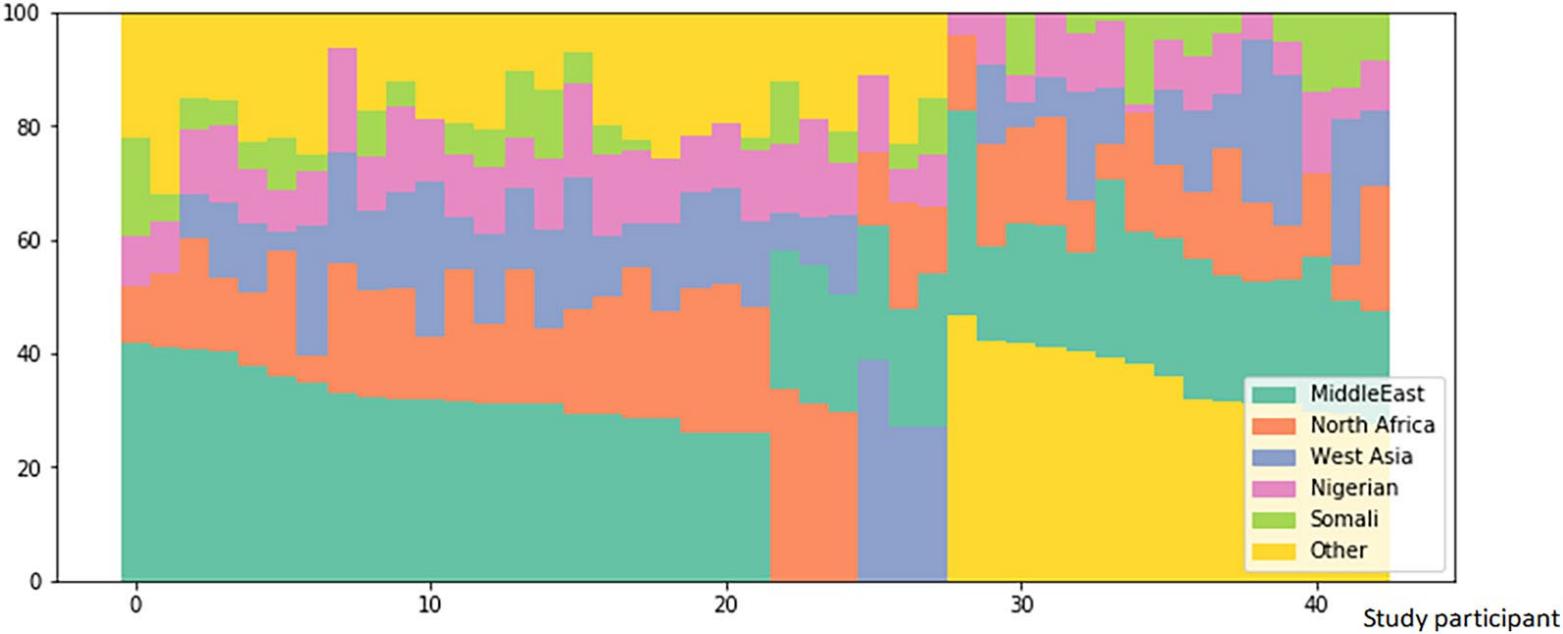
Ancestry profiles of cases and controls combined

Due to the high levels of consanguinity in the cohort resulting in long IBD segments (Fig. 1) and their distinct ethnicity profile (Fig. 2), there is no known reference panel that will share enough of their unique genomics to phase their haplotypes with high precision. The excessive resemblance between individuals gives us the ability to align haplotypes with the cohort itself as a reference panel. Therefore, for haplotype phasing, we used the Eagle V2.4 program [17] in cohort mode. We ran the DASH [18] software package with default blocks of 32 SNPs to cluster haplotypes shared between our cohort. Subsequently, we proceeded to search for specific haplotypes that were significantly associated with the patient group. Manhattan plots of the results were drawn using the Assocplots package [19].

### Statistical analysis

PLINK’s 1.9 toolset [20] permutation tests computed the *p* values. The *t* test tested for differences in the demographic characteristics between the patients and controls.

## Results

Our cohort included 14 patients and 30 controls. DNA samples were obtained from all; however, since men and women have slightly different symptom profiles [21] which could be indicative of distinct genetic risk factors, only samples from 22 female participants (11 patients and 11 controls) were evaluated for haplotype clustering and GWAS. Study participants and controls were chosen from 9 different families, which included two families where two patients were first-degree relatives. The 22 participants were similar in body mass index (BMI) (*p* = 0.64); the average BMI for the participants was 38.3 (29-59); controls, 40.4 (28-57). The patients were younger than the controls (*p* = 0.02), (average age 26.6 years; range 15–48 years); (controls, 40 years; range 13–60 years). The analysis contained 710,000 SNPs validated by MyHeritage™, of which 694,265 SNPs had a known Ref/Alt in HapMap [22].

### Clinical findings

#### Familial relationships

An analysis of the study’s cohort demonstrated a close relationship between all subjects, even those who did not identify themselves as relatives (Fig. 1).

#### Ethnicity estimate

The participants’ ethnicity was mostly African (> 30%) and Middle Eastern (30%; Fig. 2). There was no significant difference between cases and controls.

#### Association studies

Samples from the 22 female participants were evaluated in this haplotype clustering and GWAS.

#### Association to haplotypes

**Fig. 3:**
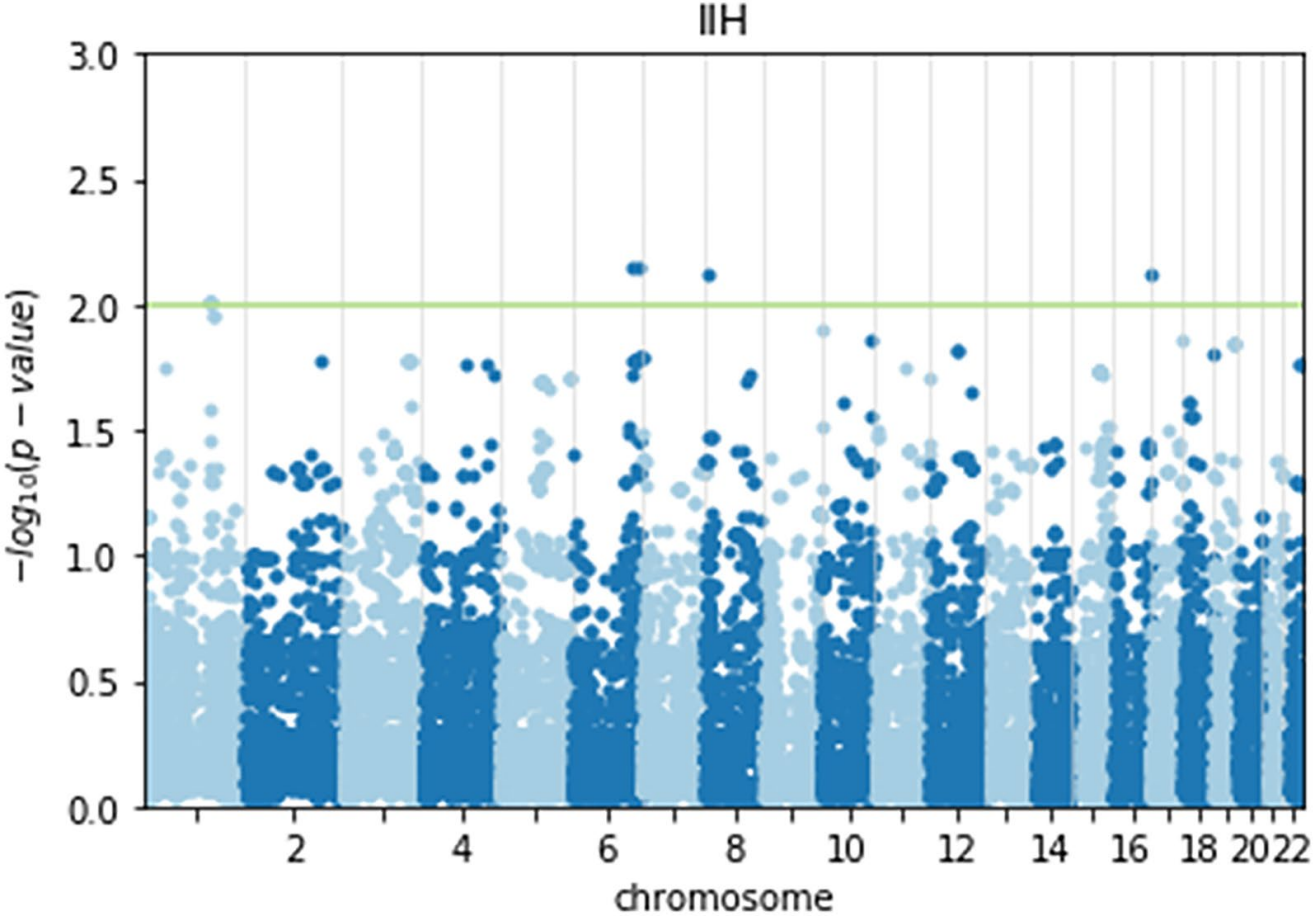
A Manhattan plot of the haplotypes’ association with IIH. The (log10) *p* values of each SNP indicating the strength of the association are plotted by chromosome from left to right. The *Y* axis corresponds to the strength of the association with the disease. The horizontal line highlights the topmost significant loci

Haplotype clustering indicated a few suspected haplotypes found significantly more prevalent in patients than in the controls. Candidate areas positively associated with IIH included genes on chromosomes 16, 8 and negatively associated with genes on chromosomes 1 and 6 (Table 1, Fig. 3). The first of these loci are located on chromosome 16 and include the *CA5A *(OMIM # 114761) and *BNP* (OMIM # 600295) genes. The second loci with a strong positive effect are on chromosome 8 and comprise too many genes. Loci with a protective effect were located on chromosome 1, and included the *PBX1* (OMIM # 176310) and *LMX1A* (OMIM # 600298) genes, and an additional locus on chromosome 6 which included the *ESR1* gene (OMIM # 133430).

**Table 1:** Haplotypes that are possibly associated with IIH

Chromosome	Start location	End location	*p* value	Genes
16	87889203	88092278	0.007502	CA5A, BANP
8	6898034	8130160	0.007468	Too many genes
1	164541977	164714538	0.009682	PBX1
1	164788638	165088116	0.009682	PBX1
1	165088116	165386158	0.009682	LMX1A
6	151677358	152054374	0.007172	ESR1

“Risk increasing” haplotypes, top part of table, “protective” haplotypes lower part of table. Uncharacterized genes and pseudogenes were omitted. Based on human genome reference sequence build version GRCh38.p14

## Discussion

The quest for understanding the genetic contributions to IIH is critical to unravel the enigmatic nature of this disorder. Our study focused on a unique Middle Eastern village with a distinct prevalence of familial ties among the IIH patients, thus providing an opportunity to explore genetic associations in the context of a homogenous population with high consanguinity rates. By employing innovative methodologies, we aimed to shed light on potential genetic factors underlying this complex condition. Our findings indicated that within this distinctive population the presence of genetic loci was possibly associated with IIH. Traditional GWAS analyzes substantial sample sizes, which can be challenging in the context of rare conditions such as IIH. Nonetheless, we adopted a creative approach by analyzing long haplotypes rather than individual SNPs, capitalizing on the fact that the patients in our cohort were related, and therefore, our results were still significant.

This approach allowed us to uncover statistically significant associations despite our limited sample size, as reported in previous studies, which have successfully demonstrated genetic associations using small cohorts, i.e., the NORDIC IIHTT Study Group who identified three candidate regions on chromosomes 5, 13, and 14 with a relatively modest sized cohort of 95 patients and controls [10]. Intriguingly, we identified regions on chromosomes 16, 8, 1, and 6 that demonstrated potential associations with IIH susceptibility or protection (Table 1). Within chromosome 16, genes CA5A and BANP surfaced as potential contributors. CA5A encodes a carbonic anhydrase enzyme [23] linked to various metabolic processes, including obesity-related pathways [24–29]. As obesity is a known risk factor for IIH, the connection between CA5A and IIH could indicate novel mechanistic pathways underlying this correlation. Interestingly, acetazolamide, the first line of drugs in treating IIH, is a carbonic anhydrase inhibitor [30]. Similarly, BANP, a tumor suppressor and cell cycle regulator [31], might play a role in IIH etiology through interactions with p53 transcription.

On chromosome 8, while the precise gene could not be pinpointed due to the region's complexity, its association with IIH remains a promising avenue for future research. Protective effects were observed on chromosomes 1 and 6, where PBX1 and LMX1A are located. PBX1 is associated with osteogenesis and insulin gene regulation [32], whereas LMX1A is linked to dopamine-producing neuron development [33], potentially implicating dopaminergic pathways in IIH. Moreover, the identification of the ESR1 gene on chromosome 6, which is linked to estrogen-regulated processes [34], adds a layer of complexity to IIH's genetic landscape. As estrogen is known to influence various physiological functions, including adipose tissue distribution, our findings could suggest novel interactions between hormonal factors and IIH susceptibility.

### Implications and future directions

Our study underscores the importance of tailoring genetic investigations to the unique characteristics of study populations. The distinctive nature of our cohort, with its high consanguinity and shared ancestry, facilitated the utilization of advanced analytical techniques, resulting in the identification of potentially relevant genetic associations. While the commonly used threshold for genome-wide association studies is 5 × 10^−8^–5 × 10^−5^, our unique cohort with familial connections led us to choose a more exploratory threshold of log10 (*p *value) of 2.0; hence, it is crucial to acknowledge the limitations of our study, primarily the modest sample size. However, we believe that our unique cohort with familial connections justifies our choice.

Our study is unique in that it focuses on a relatively rare disease, and a small, homogenous population with strong familial connections, therefore making it difficult to obtain a large sample size. Using a more exploratory threshold, we were able to identify candidate areas that may be associated with IIH in this population. In addition, we used a haplotype-based approach to estimate the associations with haplotypes instead of SNPs. Last, our pilot study aimed at identifying candidate areas that may be associated with IIH in a unique population. We acknowledge that our sample size is small and that our threshold is more exploratory than conventional thresholds. Nevertheless, we believe that our findings are promising and warrant further investigation in larger, well-phenotyped cohorts.

Several genes were identified as potential candidates associated with disease susceptibility; however, we acknowledge the speculative nature of these findings, particularly, considering our novel analytical approach. Our method introduces a new perspective, and while it holds promise, we recognize the need for caution in interpreting these preliminary associations. Despite this constraint, our approach offers a promising avenue for similar studies in other specialized cohorts with strong familial connections. As we move forward, it is imperative to expand the exploration of these loci through independent cohorts and functional studies. Future research should delve into the precise mechanisms by which these genes exert their effects in addition to investigating potential interactions with environmental factors.

## Conclusion

In conclusion, our investigation in a unique Middle Eastern village has revealed genetic loci that may contribute to IIH susceptibility and protection. Through innovative methodologies, we demonstrated the power of haplotype-based GWAS analysis in situations where traditional approaches face limitations. Our findings provide a stepping stone for future studies to further illuminate the complex genetic underpinnings of IIH and potentially offer insights into early detection and targeted interventions for individuals at risk of developing this condition. Further studies are warranted to validate and elucidate the functional significance of these findings.

## Data Availability

The datasets used and/or analyzed during the current study are available from the corresponding author on reasonable request.

## Abbreviations

SNP: Single-nucleotide polymorphisms
IIH: Idiopathic intracranial hypertension
GWAS: Genome-Wide Association Study
IIHTT: Idiopathic Intracranial Hypertension Treatment Trial
MCPH: Primary microcephaly hereditary
IBD: Identity by descent
